# Three-Dimensional Terahertz Coded-Aperture Imaging Based on Back Projection

**DOI:** 10.3390/s18082510

**Published:** 2018-08-01

**Authors:** Shuo Chen, Chenggao Luo, Hongqiang Wang, Wenpeng Wang, Long Peng, Zhaowen Zhuang

**Affiliations:** 1 School of Electronic Science, National University of Defense Technology, Changsha 410073, China; chenshuo_nudt@163.com (S.C.); penglong17@nudt.edu.cn (L.P.); zwzhuang@nudt.edu.cn (Z.Z.); 2 Science and Technology on Millimeter-Wave Laboratory, Beijing 100039, China; iyoga2017@163.com

**Keywords:** coded-aperture imaging, three-dimensional (3D), back projection (BP), synthetic aperture radar (SAR)

## Abstract

Terahertz coded-aperture imaging (TCAI) can overcome the difficulties of traditional radar in forward-looking and high-resolution imaging. Three-dimensional (3D) TCAI relies mainly on the reference-signal matrix (RSM), the large size and poor accuracy of which reduce the computational efficiency and imaging ability, respectively. According to the previous research on TCAI, traditional TCAI cannot reduce the heavy computational burden while the improved TCAI achieve reconstructing the target parts of different ranges in parallel. However, large-sized RSM still accounts for the computational complexity of traditional TCAI and the improved TCAI. Therefore, this paper proposes a more efficient imaging method named back projection (BP)-TCAI (BP-TCAI). Referring to the basic principle of BP, BP-TCAI can not only divide the scattering information in different ranges but also project the range profiles into different imaging subareas. In this way, the target parts in different subareas can be reconstructed simultaneously to synthesize the whole 3D target and thus decomposes the computational complexity thoroughly. During the pulse compression and projection processes, the signal-to-noise ratio (SNR) of BP-TCAI is also improved. This present the imaging method, model and procedures of traditional TCAI, the improved TCAI and the proposed BP-TCAI. Numerical experimental results prove BP-TCAI to be more effective and efficient than previous imaging methods of TCAI. Besides, BP-TCAI can also be seen as synthetic aperture radar (SAR) imaging with coding technology. Therefore, BP-TCAI opens a future gate combining traditional SAR and coded-aperture imaging.

## 1. Introduction

Traditional radar imaging method relies too much on the relative motion between the radar and target to achieve forward looking and staring imaging. Fortunately, terahertz coded-aperture imaging (TCAI) [1,2,3] can overcome the difficulties with a high resolution. Based on the imaging principles of both optical coded-aperture imaging [4,5] and radar coincidence imaging (RCI) [6,7], TCAI achieves reconstructing the target by producing spatiotemporal independent signals with the coded aperture. Moreover, terahertz waves (0.1–10 THz) allow visualization of hidden object at millimeter level [8,9,10] with stronger penetration capability than light and higher resolution over microwave.

Because of the promising ability in flexible manipulation on terahertz and millimeter waves, metasurfaces have been applied into areas of high-resolution computational imaging [11,12] and some scanning devices [13,14]. Besides, the Harvard Robotics Laboratory (HRL) manufactures an economic high-resolution coded aperture subreflector array (CASA) to see weapons or explosives concealed on a person [15]. Therefore, metasurfaces open bright prospects for device support of ceded aperture.

Because the scale of the reference signal matrix is proportional to the number of resolution cell, the computational complexity increases significantly for 3D target in terahertz band. Moreover, traditional TCAI [16] algorithms usually fail to resolve low signal-to-noise ratio (SNR) targets. To solve the problems, Ref. [17] proposes an imaging method to obtain the range profile with matched filtering, which belongs to pulse compression. However, when the SNR is under -10dB, pulse compression has new problem of extracting the true target positions form the range profile and thus results in the imaging failure. Therefore, Ref. [18] tries to get the right positions of the spike pulses by learning the intrinsic nature of the range profile with geometric measures (GM) [19,20]. Ref. [18] attempts to introduce the GM or the information geometry into radar imaging area. Until now, TCAI has two kinds of imaging methods: time-domain TCAI, range-domain TCAI. Ref. [16] and traditional TCAIs are time-domain TCAIs while Refs. [17,18] belong to range-domain TCAI.

However, when the imaging plane is too large or the resolution cell is too small, the computational burden is still pretty heavy for traditional TCAI and GM-TCAI. Besides, both traditional TCAI and GM-TCAI modulate the signals in the transmitting terminal, which terribly reduce the working distance. Therefore, based on BP [21,22] and single input multiple output (SIMO) architecture, this paper proposes a new imaging method called back projection (BP)-TCAI (BP-TCAI). Different from current TCAI, the SIMO architecture performs the modulation operation in the receiving terminal with multiple detectors, which has no influence on the working distance. More importantly, BP projects the received time-domain signals to space domain, where the signals corresponding to different subareas of the same imaging plane are further divided. Therefore, the target parts of different subareas can be reconstructed independently and simultaneously with high SNR and finally are combined together to construct the whole 3D target. Because the adopted echo signals for imaging are located in space domain, the BP-TCAI can be categorized as a kind of space-domain TCAI.

This paper is organized as follows. Section 2.1 and Section 2.2 present the basic imaging model and procedure of traditional TCAI and GM-TCAI, respectively. Section 2.3 introduces the detailed imaging method, model and procedure of the proposed BP-TCAI. In Section 3, numerical experiments demonstrate the superiority of BP-TCAI over traditional TCAI and GM-TCAI for low SNR 3D Targets. Finally, we summarize the main advantages of BP-TCAI and conclude with the future development of BP-TCAI in Section 4.

## 2. Imaging Method

### 2.1. Traditional TCAI

**Figure 1:**
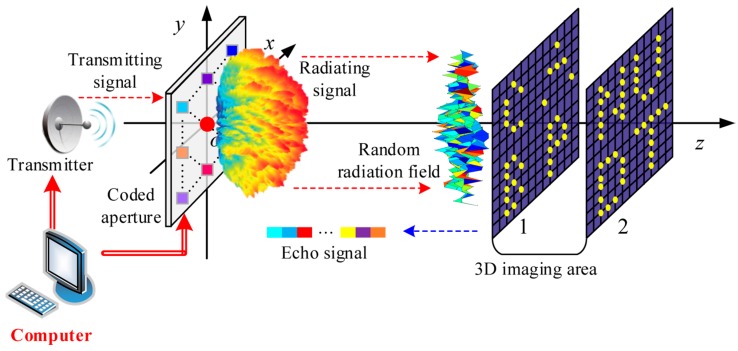
Schematic diagram of traditional 3D TCAI architecture.

The traditional 3D TCAI in Figure 1 features with a transmitter, a coded aperture and a computer. The signal propagation includes the transmitting and receiving processes, which are marked as red and blue dashed lines. Firstly, the coded aperture randomly modulates the amplitude or phase of the transmitting signal for arbitrary measurement modalities. On the coded aperture, different colors denote different amplitude or phase modulation. Secondly, the radiating signal through the coded aperture convolves with the 3D target. The 3D imaging area is subdivided into tiny grid cells according to the imaging resolutions in range, azimuth and elevation directions. It is assumed that the scatters are at the centers of the grid cells. Shown as a filled red circle on the coded aperture, the single detector receives the echo signal carrying target information. Eventually, the detector transfers the echo signal to the computer for imaging. Besides, the computer can control both the transmitter and the coded aperture. To be clear, we present the signal propagation and imaging model of traditional TCAI further in Section 2.1.1 and Section 2.1.2. For simplicity, we suppose the coded aperture only modulates the signal phase instead of the amplitude.

#### 2.1.1. Signal Propagation

The transmitting signal before modulation is linear frequency modulation (LFM) signal and it is formulated as:

(1) $$s_{t}(t)=\exp\left[j2\pi f\cdot t\right]\;$$

where $s_{t}(t)$ is the transmitting signal at time t, f is the signal frequency and j denotes the imaginary unit. Besides, $f=f_{0}+0.5\gamma t$, where $f_{0}$ and γ are the center frequency and chirp rate, respectively.

The signal arriving at the coded aperture is assumed as plane wave. Therefore, the time delay terms for each transmitting element of the coded aperture are the same and they can be set as zeroes. As the coded aperture contains *I* transmitting elements, the radiating signal after modulation is deduced as:

(2) $$s_{c}(t)=\sum_{i=1}^{I}\exp\left[j2\pi f\cdot t+\varphi_{i}\left(t\right)\right]\;$$

where $\varphi_{i}\left(t\right)$ is the random phase-modulation term for the *i*-th transmitting element at time *t*. $\varphi_{i}\left(t\right)$ changes randomly over fast time within the range of $\left[p_{l},p_{h}\right]$, where $p_{l}$ and $p_{h}$ are the minimum and maximum value of $\varphi_{i}\left(t\right)$. Phase modulation range is proportional to high-resolution imaging, while excessive modulation will damage the beam forming. $\left[p_{l},p_{h}\right]$ should be set as proper value to strike a balance of high-resolution imaging and beam forming. Then, the radiating signal illustrates the 3D imaging area. High-resolution imaging relies on strong spatiotemporal independence of the radiation field on the 3D imaging area. Therefore, we hope to improve the phase modulation degree for high-resolution imaging. However, excessive modulation of traditional TCAI will damage the beam forming and thus reduce the imaging distance. The proposed BP-TCAI achieves better imaging performance with no requirements of shortening the imaging distance, which will be further described in Section 2.3.

Reflected by the 3D target, the echo signal arriving at the single detector is written as:

(3) $$s_{r0}(t)=\sum_{k=1}^{K}\sum_{i=1}^{I}\beta_{k}\cdot \exp\left[j2\pi f\left(t-t_{i,k,o}\right)+\varphi_{i}\left(t\right)\right]\;$$

where $\beta_{k}$ is the scattering coefficient corresponding to the *k*-th grid-cell, $t_{i,k,o}$ is the total time delay passing though the *i*-th transmitting element, the *k*-th grid cell and the receiver. *K* is the grid-cell number of the 3D imaging area.

Apparently, the echo signal cannot be sampled directly at THz band. The same as the transmitting signal written in Equation (1), the reference signal is also generated for sampling the echo signal. By mixing the echo signal in Equation (3) with the reference signal, the sampled signal is formulated as

(4) $$s_{r}(t)=\sum_{k=1}^{K}\sum_{i=1}^{I}\beta_{k}\cdot \exp\left[-j2\pi ft_{i,k,o}+\varphi_{i}\left(t\right)\right]\;$$

Compared with Equation (3), the item of exp(j2πft) is eliminated. Therefore, the echo signal is down converted into baseband signal and it can be sampled directly in a general way.

#### 2.1.2. Imaging Model

Based on time discretion of Equation (4), the mathematical model of traditional TCAI is deduced as:

(5) $$\begin{array}{l} Sr=S\cdot \beta +w\\ \left[\begin{array}{c} s_{r}\left(t_{1}\right)\\ s_{r}\left(t_{2}\right)\\ \ldots \\ s_{r}\left(t_{N}\right) \end{array}\right]=\left[\begin{array}{cccc} s\left(t_{1},1\right) & s\left(t_{1},2\right) & \ldots & s\left(t_{1},K\right)\\ s\left(t_{2},1\right) & s\left(t_{2},2\right) & \ldots & s\left(t_{2},K\right)\\ \ldots & \ldots & \ldots & \ldots \\ s\left(t_{N},1\right) & s\left(t_{N},2\right) & \ldots & s\left(t_{N},K\right) \end{array}\right]\cdot \left[\begin{array}{c} \beta_{1}\\ \beta_{2}\\ \ldots \\ \beta_{K} \end{array}\right]+\left[\begin{array}{c} w_{1}\\ w_{2}\\ \ldots \\ w_{N} \end{array}\right] \end{array}\;$$

where Sr and S are the echo vector (EV) and reference-signal matrix (RSM), respectively. β is the scattering-coefficient vector (SCV). w is the measurement noise vector (MNV). *N* and *K* are the sampling-time and grid-cell numbers, respectively. $s\left(t_{n},k\right)$, the array element of S, is formulated as:

(6) $$s\left(t_{n},k\right)=\sum_{i=1}^{I}\beta_{k}\cdot \exp\left[-2\pi ft_{i,k,o}+\varphi_{i}\left(t_{n}\right)\right]\;$$

where $t_{n}$ denotes the *n*-th sampling time.

Table 1 has shown the imaging procedure of traditional TCAI as below.

### 2.2. GM-Based TCAI

With the knowledge of solving linear equations, Equation (5) can be solved by compressed sensing (CS) algorithm. However, when the SNR is too low, the 3D target becomes difficult to be reconstructed in time domain. Due to large amount of meshed grid cells, the large-scaled RSM results in heavy computational burden. To solve problems of low SNR and increasing computational complexity, the improved TCAIs proposed in Refs. [19,20] transforms the EV and RSM into range domain. Essentially, the transformation is the process of pulse compression, which can improve the SNR in a certain degree and distinguish the target information in different ranges [17]. However, the range information is difficult to be detected when the SNR is under −10 dB. Therefore, GM-TCAI proposed in Ref. [18] adopts GM to find the range cells containing scattering information. Both Refs. [17,18] belong to range-domain TCAIs while Ref. [18] is an improved version of Ref. [17]. Therefore, this paper only shows the basic imaging procedures and model of GM-TCAI and more details are presented in Ref. [18].

#### 2.2.1. EV Extraction of GM-TCAI

As we know, both matched filtering and dechirping belong to pulse compression. Matched filtering is a more general way of pulse compression designed for common signals, where the correlation is utilized. Because of the particular characteristics, LFM signal can also be compressed by dechirping, which obtain the range by processing the baseband echo signal with Fourier transformation. Because the transmitting signal is LFM signal, the baseband echo signal in Equation (5) can be compressed by simple Fourier transformation. Then, the echo after pulse compression is shown as:

(7) $$S_{r}(f_{t})=F\left[s_{r}\left(t\right)\right]\;$$

where F(⋅) denotes the Fourier transformation, which can be operated fast with current computational technology. $S_{r}(f_{t})$ is the echo in frequency domain. To avoid confusion of the signal frequency, we adopt $f_{t}$ to describe the frequency symbol corresponding to *t*. $S_{r}(f_{t})$ presents spike pulses in the range cells containing target information. The scattering information within the same range gathers in the same spike pulse. However, when the SNR is too low, the target-containing range cells are difficult to recognize. Ref. [19] adopts GM to project $S_{r}(f_{t})$ into manifold and find the useful range cells by Kullback–Leibler divergence (KLD) [17,18,19]. Detailed detecting procedure of GM-TCAI has been presented enough in Ref. [19]. Herein, FSr is defined as the original EV of GM-TCAI, which is obtained by frequency discretion of $S_{r}(f_{t})$.

**Figure 2:**
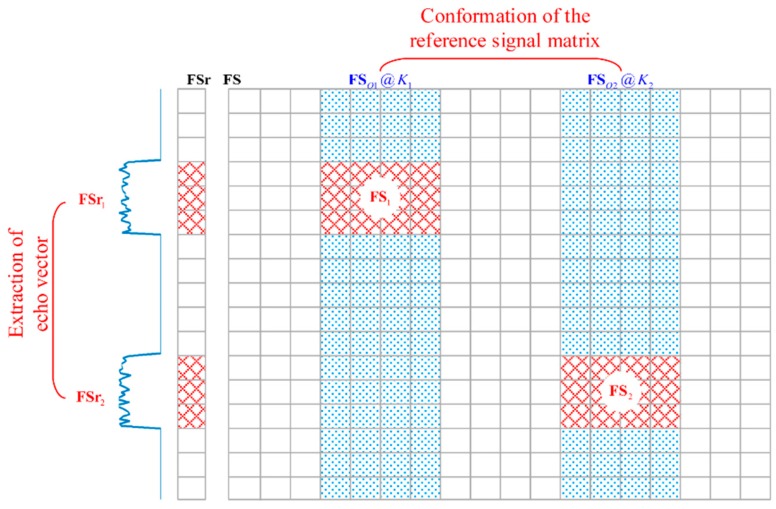
Extraction of the echo vector (EV) and conformation of the reference-signal matrix (RSM) for geometric measures (GM)-TCAI.

The 3D imaging area in Figure 1 has two imaging planes in different ranges. As each imaging plane is about in one range cell, FSr will show two spike pulses. Thus, $FSr_{1}$ and $FSr_{2}$ can be extracted from FSr, which is shown in Figure 2. $FSr_{1}$ and $FSr_{2}$ are two GM-TCAI EVs related to the two spike pulses. Besides, we adopt $r_{1}$ and $r_{2}$ to index the corresponding row positions of $FSr_{1}$ and $FSr_{2}$ in FSr. $K_{1}$ and $K_{2}$ are the grid-cell numbers of the two imaging planes, respectively.

#### 2.2.2. RSM Conformation of GM-TCAI

As shown in Figure 2, FS is the RSM in parallel with the original EV FSr. Extracted partly from $FS_{{}_{1}}^{o}$ and $FS_{{}_{2}}^{o}$, $FS_{1}$ and $FS_{2}$ are the corresponding RSMs of $FSr_{1}$ and $FSr_{2}$, separately. Moreover, $FS_{{}_{1}}^{o}$ and $FS_{{}_{2}}^{o}$ are obtained by extracting corresponding columns of FS. Similar to FSr, each column of FS is processed with Fourier transformation as:

(8) $$S(f_{t},k)=F\left[s\left(t,k\right)\right]\;$$

where $S(f_{t},k)$ is the Fourier transformation of s(t,k). By frequency discretion on $S(f_{t},k)$, we get the *k*-th column of FS.

Therefore, $FS_{1}$ and $FS_{2}$ are constructed by extracting corresponding rows of $FS_{{}_{1}}^{o}$ and $FS_{{}_{2}}^{o}$, which are deduced by combining the related $K_{1}$ and $K_{2}$ columns of FS.

#### 2.2.3. Imaging Model of GM-TCAI

Taken an imaging plane named *x* for example, the imaging model of GM-TCAI is deduced as:

(9) $$\begin{array}{l} FSr_{x}=FS_{x}\cdot \beta_{x}+w_{x}^{GM}\\ \left[\begin{array}{c} Sr\left(f_{1}\right)\\ Sr\left(f_{2}\right)\\ \ldots \\ Sr\left(f_{N_{x}}\right) \end{array}\right]=\left[\begin{array}{cccc} S\left(f_{1},1\right) & S\left(f_{1},2\right) & \ldots & S\left(f_{1},K_{x}\right)\\ S\left(f_{2},1\right) & S\left(f_{2},2\right) & \ldots & S\left(f_{2},K_{x}\right)\\ \ldots & \ldots & \ldots & \ldots \\ S\left(f_{N_{x}},1\right) & S\left(f_{N_{x}},2\right) & \ldots & S\left(f_{N_{x}},K_{x}\right) \end{array}\right]\cdot \left[\begin{array}{c} \beta_{1}\\ \beta_{2}\\ \ldots \\ \beta_{K_{x}} \end{array}\right]+\left[\begin{array}{c} w_{1}^{GM}\\ w_{2}^{GM}\\ \ldots \\ w_{N_{x}}^{GM} \end{array}\right] \end{array}$$

where $FSr_{x}$, $FS_{x}$, $\beta_{x}$ and $w_{x}^{GM}$ are the EV, RSM, SCV and MNV of GM-TCAI, respectively. $N_{x}$ and $K_{x}$ are the extracted-frequency and grid-cell numbers of imaging plane *x*, respectively.

Based on Equation (9), each imaging plane in Figure 1 can be reconstructed in parallel to decompose the global computational complexity and then are combined together to reconstruct the 3D target. Table 2 has shown the imaging procedure of GM-TCAI as below.

### 2.3. BP-Based TCAI

When the imaging plane is too large or the resolution cell is too small, the computational burden is still pretty heavy for traditional TCAI and GM-TCAI. This paper proposes an effective and efficient TCAI method based on BP. In this approach, BP helps to transforms the coding imaging from time domain into space domain, where different target parts in different sub-areas can be reconstructed independently and simultaneously with high SNR. There are two factors that can improve the SNR of BP-TCAI. On one hand, the echo signal of the *i*-th receiver is transformed from time domain into range profiles via Equation (13). As shown in Section 2.2, the SNR can be improved by extracting the range cells containing scattering information. On the other hand, BP helps project the range-domain signals into space domain. Thus, the space areas containing target information can be extracted for further imaging and thus improve the SNR again.

**Figure 3:**
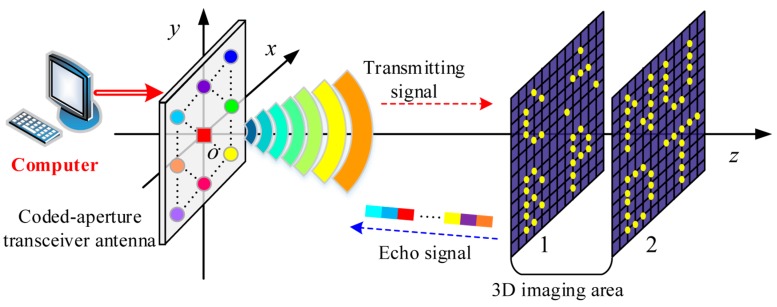
Schematic diagram of 3D back projection (BP)-TCAI architecture.

As shown in Figure 3, the BP-TCAI is mainly composed of a coded-aperture transceiver antenna and a computer. Similar to Figure 1, the red and blue dashed lines denote the transmitting and receiving processes, respectively. Unlike the traditional TCAI architecture, the proposed BP-TCAI is a SIMO system, which denotes a single transmitting antennas and multiple receiving antennas. The red square in the center of the coded aperture denotes the single transmitter. The colorful circles describing the multiple receivers are located in the coded aperture in array form. Different colors mean different amplitude or phase modulation. Different from the traditional TCAI, the modulation operation is achieved in the receiving terminal with no requirements of reducing the working distance. The computer can control the single transmitter to send signal and the multiple receivers to receive the echo added with modulation. Finally, the computer processes all the echo signals for high-resolution BP-TCAI. The detailed signal propagation process of BP-TCAI is shown as below. Similar to Section 2.1, we suppose the coded aperture only modulates the signal phase rather than the amplitude.

Firstly, the transmitting signal from the single transmitter illustrates the 3D imaging area directly. The signal form is the same as that in Equation (1).

Secondly, convolved with the 3D target, the radiating signal is deduced as:

(10) $$S_{rad}^{BP}(t)=\sum_{k=1}^{K}\beta_{k}\cdot \exp\left[j2\pi f\left(t-t_{o,k}\right)\right]\;$$

where $S_{rad}^{BP}(t)$ is the radiating signal at time t, $t_{o,k}$ is time delay between the transmitter and the *k*-th grid cell.

Thirdly, the echo signal arriving at the *i*-th coded-aperture receiver is written as:

(11) $$S_{r0_{i}}^{BP}(t)=\sum_{k=1}^{K}\beta_{k}\cdot \exp\left[j2\pi f\left(t-t_{o,k,i}\right)+\varphi_{t,i}\right]\;$$

where $\varphi_{t,i}$ is the random phase-modulation term for the *i*-th coded-aperture receiver at time *t*, $t_{o,k,i}$ is the total time delay though the single transmitter, the *k*-th grid cell and the *i*-th coded-aperture receiver.

Finally, the baseband echo signal is sampled by mixing $S_{r0_{i}}^{BP}(t)$ with the reference signal. The reference signal is the same as Equation (1), while the baseband echo signal is formulated as,

(12) $$S_{r_{i}}^{BP}(t)=\sum_{k=1}^{K}\beta_{k}\cdot \exp\left[-j2\pi ft_{o,k,i}+\varphi_{i}\left(t\right)\right]\;$$

Similar to Equation (5), $S_{r_{i}}^{BP}(t)$ can be sampled directly at baseband frequency.

Moreover, the imaging model and procedures are illustrated in detail as below.

#### 2.3.1. EV Extraction of BP-TCAI

According to the BP theory in SAR imaging [21,22], the target scattering information is obtained by projecting the range profile to the space position of each resolution cell. The resolution cell denotes the grid cell in coded-aperture imaging.

Firstly, similar to the principle of Equation (7), the range profile of $S_{r_{i}}^{BP}(t)$ is deduced from:

(13) $$IFS_{r_{i}}^{BP}(f_{t})=\mathrm{IF}\left(S_{r_{i}}^{BP}(t)\right)\;$$

where $IFS_{r_{i}}^{BP}(f_{t})$ is the inverse Fourier transformation (IFT) of $S_{r_{i}}^{BP}(t)$, IF(⋅) denotes the IFT operation.

Then, the scattering coefficient of the *k*-th grid cell is deduced from:

(14) $$\begin{array}{cc} BS_{r}\left(s_{k}\right) & =\sum_{i=1}^{I}IFS_{r_{i}}^{GM}(f_{t})\cdot \exp\left(j2\pi f_{c}t_{o,k,i}\right)\\ & =\sum_{i=1}^{I}IFS_{r_{i}}^{GM}(f_{t})\cdot \exp\left(j\varphi_{k,i}\right) \end{array}\;$$

where $\varphi_{k,i}=2\pi f_{c}t_{o,k,i}$ is the phase compensation term corresponding to the *k*-th grid cell and the *i*-th coded-aperture receiver. $f_{c}$ is the center frequency of the transmitting signal. $BS_{r}\left(s_{k}\right)$ is the scattering coefficient of the *k*-th grid cell. Apparently, $BS_{r}\left(s_{k}\right)$ is obtained by coherent superposition of all the *I* range profiles, which is deduced from Equation (11). $s_{k}$ describes the *k*-th space-domain frequency of $f_{t}$. Therefore, the grid cell containing target information can be extracted for further imaging.

As described in Section 2.2.3, the imaging plane *x* contains $K_{x}$ grid cells. Therefore, we combine the $K_{x}$ scattering coefficients $BS_{r}\left(s_{k_{x}}\right),k_{x}=1,\cdots K_{x}$ together to construct the EV of BP-TCAI, which is written as:

(15) $$BSr_{x}={\left[BS_{r}\left(s_{k_{1}}\right),\cdots ,BS_{r}\left(s_{k_{x}}\right),\cdots ,BS_{r}\left(s_{K_{x}}\right)\right]}^{T}\;$$

where ${\left[\cdot \right]}^{T}$ describes the transposition of vector or matrix.

**Figure 4:**
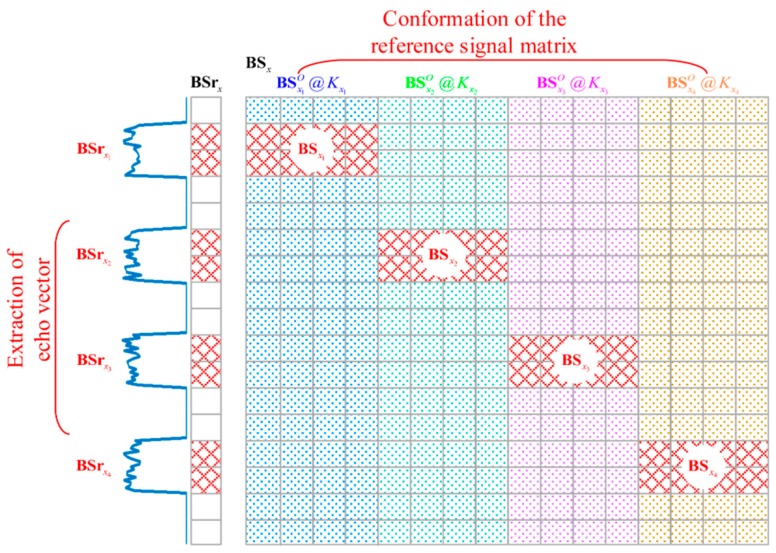
Extraction of the EV and conformation of the RSM for BP-TCAI.

As shown in Figure 3, each imaging plane is subdivided into four subareas, each of which has a character-shaped target. The subareas of imaging plane *x* are named as $x_{1}$, $x_{2}$, $x_{3}$ and $x_{4}$ respectively. Thus, the stronger scattering coefficients are extracted independently from $BSr_{x}$ to construct $BSr_{x_{1}}$, $BSr_{x_{2}}$, $BSr_{x_{3}}$ and $BSr_{x_{4}}$,which are shown in Figure 4. Besides, $r_{x_{1}}$, $r_{x_{2}}$, $r_{x_{3}}$ and $r_{x_{4}}$ are indexed as the corresponding row positions of $BSr_{x_{1}}$, $BSr_{x_{2}}$, $BSr_{x_{3}}$ and $BSr_{x_{4}}$ in $BSr_{x}$. $K_{x_{1}}$, $K_{x_{2}}$, $K_{x_{3}}$ and $K_{x_{4}}$ are the grid-cell numbers of the subareas, respectively.

#### 2.3.2. RSM Conformation of BP-TCAI

As shown in Figure 4, $BS_{x}$ is the BP-based RSM corresponding to the EV $BSr_{x}$. $BS_{x_{1}}^{O}$, $BS_{x_{2}}^{O}$, $BS_{x_{3}}^{O}$ and $BS_{x_{4}}^{O}$ are equally divided from $BS_{x}$. The column numbers of $BS_{x_{1}}^{O}$, $BS_{x_{2}}^{O}$, $BS_{x_{3}}^{O}$ and $BS_{x_{4}}^{O}$ are $K_{x_{1}}$, $K_{x_{2}}$, $K_{x_{3}}$ and $K_{x_{4}}$, respectively. The row numbers of $BS_{x_{1}}^{O}$, $BS_{x_{2}}^{O}$, $BS_{x_{3}}^{O}$ and $BS_{x_{4}}^{O}$ are the same as $BSr_{x}$. As described in Section 2.2.1, $r_{x_{1}}$, $r_{x_{2}}$,$r_{x_{3}}$ and $r_{x_{4}}$ are the row-position tags of $BSr_{x_{1}}$, $BSr_{x_{2}}$, $BSr_{x_{3}}$ and $BSr_{x_{4}}$ in $BSr_{x}$. Thus, $r_{x_{1}}$, $r_{x_{2}}$,$r_{x_{3}}$ and $r_{x_{4}}$ are used to extract corresponding rows of $BS_{x_{1}}^{O}$, $BS_{x_{2}}^{O}$, $BS_{x_{3}}^{O}$ and $BS_{x_{4}}^{O}$ and eventually obtain the required RSMs $BS_{x_{1}}$, $BS_{x_{2}}$, $BS_{x_{3}}$ and $BS_{x_{4}}$, which match with $BSr_{x_{1}}$, $BSr_{x_{2}}$, $BSr_{x_{3}}$ and $BSr_{x_{4}}$, respectively.

Apparently, $BS_{x}$ should be deduced to obtain $BS_{x_{1}}$, $BS_{x_{2}}$, $BS_{x_{3}}$ and $BS_{x_{4}}$. The detailed deduction process is shown as below.

Firstly, we define the reference signal related to the *i*-th coded-aperture detector and the $k_{x}$-th grid cell as:

(16) $$S_{i}^{BP}\left(t,k_{x}\right)=\beta_{k}\cdot \exp\left[-\left(j2\pi f\left(t-t_{o,k,i}\right)+\varphi_{t,i}\right)\right]\;$$

Referring to Equations (11)–(13), the $k_{x}$-th column of $BS_{x}$ is formulated as:

(17) $$IFS_{i}^{BP}(f_{t},k_{x})=\mathrm{IF}\left(S_{i}^{BP}(t,k_{x})\right)\;$$

(18) $$\begin{array}{cc} BS\left(s_{k_{m}},k_{x}\right) & =\sum_{i=1}^{I}IFS_{i}^{BP}(f_{t},k_{x})\cdot \exp\left(j2\pi f_{c}t_{o,k_{m},i}\right)\\ & =\sum_{i=1}^{I}IFS_{i}^{BP}(f_{t},k_{x})\cdot \exp\left(j\varphi_{k_{m},i}\right) \end{array}\;$$

(19) $$BS_{k_{x}}={\left[BS\left(s_{1},k_{x}\right),\cdots ,BS\left(s_{k_{x}},k_{x}\right),\cdots ,BS\left(s_{K_{x}},k_{x}\right)\right]}^{T}\;$$

where $IFS_{i}^{BP}(f_{t},k_{x})$ is the inverse Fourier transformation (IFT) of $S_{i}^{BP}(t,k_{x})$, $BS\left(s_{k_{m}},k_{x}\right)$ is the $k_{m}$-th vector element of $BS_{k_{x}}$ and $BS_{k_{x}}$ is the $k_{x}$-th column of $BS_{x}$. Moreover, $k_{m}=1,2,\cdots ,K_{x}$, $\varphi_{k_{m},i}=2\pi f_{c}t_{o,k_{m},i}$ is the phase compensation term corresponding to the $k_{m}$-th grid cell and the *i*-th coded-aperture receiver. $t_{o,k_{m},i}$ is the total time delay through the single transmitter, the $k_{m}$-th grid cell and the *i*-th coded-aperture receiver.

Eventually, $BS_{x}$ is formulated as:

(20) $$BS_{x}=\left[BS_{1},\cdots ,BS_{k_{x}},\cdots ,BS_{K_{x}}\right]\;$$

Therefore, $BS_{x_{1}}^{O}$, $BS_{x_{2}}^{O}$, $BS_{x_{3}}^{O}$ and $BS_{x_{4}}^{O}$ are divided from $BS_{x}$. Furthermore, $BSr_{x_{1}}$, $BSr_{x_{2}}$, $BSr_{x_{3}}$ and $BSr_{x_{4}}$ are extracted from $BS_{x_{1}}^{O}$, $BS_{x_{2}}^{O}$, $BS_{x_{3}}^{O}$ and $BS_{x_{4}}^{O}$ with corresponding row-position tags.

#### 2.3.3. Imaging Model of BP-TCAI

A subarea subdivided from imaging plane *x* is named as $x_{a}$. The imaging model of BP-TCAI for $x_{a}$ is deduced as:

(21) $$\begin{array}{l} BSr_{x_{a}}=BS_{x_{a}}\cdot \beta_{x_{a}}+w_{x_{a}}^{BP}\\ \left[\begin{array}{c} BSr\left(s_{1}\right)\\ BSr\left(s_{2}\right)\\ \ldots \\ SSr\left(s_{{N_{x}}_{a}}\right) \end{array}\right]=\left[\begin{array}{cccc} BS\left(s_{1},1\right) & BS\left(s_{1},2\right) & \ldots & BS\left(s_{1},K_{x_{a}}\right)\\ BS\left(s_{2},1\right) & BS\left(s_{2},2\right) & \ldots & BS\left(s_{2},K_{x_{a}}\right)\\ \ldots & \ldots & \ldots & \ldots \\ BS\left(s_{{N_{x}}_{a}},1\right) & BS\left(s_{{N_{x}}_{a}},2\right) & \ldots & BS\left(s_{{N_{x}}_{a}},{K_{x}}_{a}\right) \end{array}\right]\cdot \left[\begin{array}{c} \beta_{1}\\ \beta_{2}\\ \ldots \\ \beta_{{K_{x}}_{a}} \end{array}\right]+\left[\begin{array}{c} w_{1}^{BP}\\ w_{2}^{BP}\\ \ldots \\ w_{{N_{x}}_{a}}^{BP} \end{array}\right] \end{array}$$

where $SSr_{x_{a}}$, $SS_{x_{a}}$, $\beta_{x_{a}}$ and $w_{x_{a}}^{BP}$ are the EV, RSM, SCV and MNV of BP-TCAI, separately. $N_{x_{a}}$ and $K_{x_{a}}$ are the extracting and the entire grid-cell numbers of subarea $x_{a}$, respectively.

According to Equation (19), subareas of each imaging plane in Figure 3 are resolved in parallel and then are synthesized together to get the 3D target. In this method, both the computational complexity and noise power are much less than the traditional TCAI and GM-TCAI. Table 3 has shown the imaging procedure of BP-TCAI as below.

### 2.4. Comparisons of Computational Complexity

As for the computational complexity comparisons, the most time-consuming part are the reconstruction algorithms according to Equations (4), (9) and (21) for traditional TCAI, GM-TCAI and BP-TCAI, respectively. For example, SBL is adopted as the reconstruction algorithm for all the three imaging methods. Furthermore, the most time-consuming steps of SBL are matrix inversion and matrix-vector multiplication, the costs of the two operations are $O\left(K^{3}\right)$ and $O\left(K^{2}\right)$, respectively. *K* is the number of the grid cell. As shown in Equations (4), (9) and (21), the grid-cell numbers for traditional TCAI, GM-TCAI and BP-TCAI are K, $K_{x}$ and $K_{x_{a}}$, respectively. Besides, the number of iteration for SBL can be set as M. Therefore, for the reconstruction part, the computational complexities of the three imaging methods are $O\left(M\left(K^{3}+K^{2}\right)\right)$, $O\left(M\left(K_{x}{}^{3}+K_{x}{}^{2}\right)\right)$ and $O\left(M\left(K_{x_{a}}{}^{3}+K_{x_{a}}{}^{2}\right)\right)$, separately. Because of the extracting processing, $K_{x_{a}}$ is much less than K and $K_{x}$. Therefore, the computational burden of BP-TCAI is reduced more significantly than traditional TCAI and GM-TCAI. Besides the reconstruction algorithm, GM-TCAI and BP-TCAI transform the received time-domain signals into range profiles and space domain according to Equations (7) and (14), separately. The transformation complexities of GM-TCAI and BP-TCAI can be calculated as O(KlogK) and O(IK), where I is the number of coded-aperture receivers. Apparently, compared with the reconstruction part, the transformation step of GM-TCAI and BP-TCAI only cost little time. From the theoretical analysis, it can be concluded that BP-TCAI consumes much less time than traditional TCAI and GM-TCAI.

## 3. Experimental Results

In this section, firstly, we analyze the point spread function (PSF) of BP-TCAI with different phase-modulation degrees. Essentially, the PSF is the imaging result of a point target located in the center of an imaging plane. Secondly, the range profiles under different SNRs are presented to detect the locations of the two imaging planes. Besides, to find the influence of phase modulation on the BP imaging, we present the BP projecting results without and with phase modulation, respectively. Finally, sparse Bayesian learning (SBL) [23] is used to compare the imaging results of traditional TCAI, GM-TCAI and BP-TCAI. Besides, both traditional TCAI and GM-TCAI calculate the radiating signals and echo signals according to Equations (2) and (6), respectively. Besides, BP-TCAI calculate the radiating and echo signals according to Equations (10) and (12), respectively.

Moreover, to evaluate the imaging performance of TCAI, GM-TCAI and BP-TCAI, we adopt the relative imaging error (RIE) and probability of successful imaging (PSI) [24], which are defined as

(22) $$\mathrm{RIE}≜20\log_{10}\left({\left\|\hat{\beta }-\beta \right\|}_{2}/{\left\|\beta \right\|}_{2}\right)\;$$

(23) $$\mathrm{PSI}≜20\log_{10}\left(\min{\left(\hat{\beta }\right)}_{\Lambda }/\max{\left(\bar{\hat{\beta }}\right)}_{\Lambda }\right)\;$$

where β and $\hat{\beta }$ are the true and estimated SCVs, respectively. ${\left(\hat{\beta }\right)}_{\Lambda }$ contains the values that $\hat{\beta }$ carries at the correct basis set Λ. ${\left(\bar{\hat{\beta }}\right)}_{\Lambda }$ takes 0 at Λ and takes the same values as $\hat{\beta }$ at every other indices. RIE is inverse proportional to the imaging quality while PSI is proportional to the imaging performance.

The primary parameters used in the simulations are given in Table 4. As shown in Figure 3, the 3D imaging area has two imaging planes, each of which contains four subareas. Traditional TCAI and GM-TCAI requires random phase modulation while it may damage the beam formation and thus reduce the maximum imaging range. BP-TCAI applies the modulation operation into the receiving terminal, which has no influence on the original imaging distance. Besides, the parameters of the coded aperture for traditional TCAI, GM-TCAI and BP-TCAI are the same. Unlike TCAI and GM-TCAI, the coded aperture of BP-TCAI functions as a SIMO system. The experiments are performed on a computer with Intel Core CPU i7-8700U at 3.2 GHz and 16 GB of memory.

### 3.1. PSF Analysis

**Figure 5:**
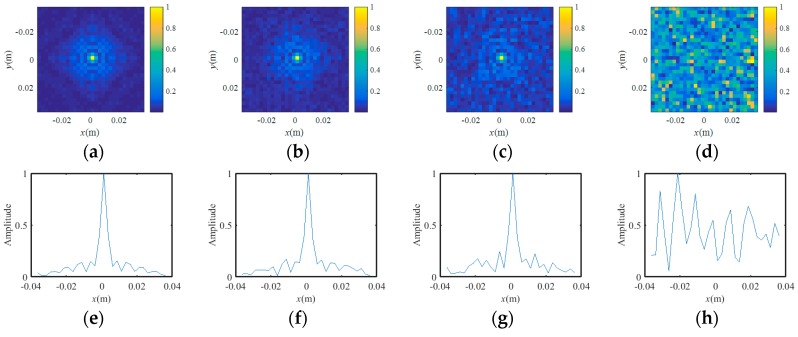

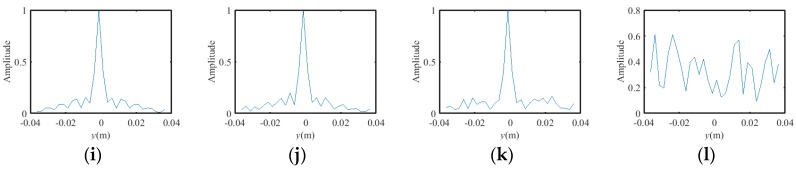
Vertical view of the point spread function (PSF) with different phase-modulation ranges, including, (**a**) no modulation, (**b**) [−0.25π, 0.25π], (**c**) [−0.5π, 0.5π], (**d**) [−π, π], respectively. The x-axis cross-section view of the PSF function (PSF) with different phase-modulation ranges, including, (**e**) no modulation, (**f**) [−0.25π, 0.25π], (**g**) [−0.5π, 0.5π], (**h**) [−π, π], respectively. The y-axis cross-section view of the PSF function (PSF) with different phase-modulation ranges, including, (**i**) no modulation, (**j**) [−0.25π, 0.25π], (**k**) [−0.5π, 0.5π], (**l**) [−π, π], respectively.

As we know, traditional BP imaging for SAR or inverse synthetic aperture imaging (ISAR) is performed without phase modulation, which is the guarantee of successful imaging for coded-aperture imaging. The BP-TCAI method can also be seen as coding-array SAR. To analyze the imaging performance of coding-array SAR, Figure 5 presents the PSF with different modulation degrees, including no modulation, [−0.25π, 0.25π], [−0.5π, 0.5π] and [−π, π] continuous phase modulations. The PSF presented in Figure 5 is simply the projection results of BP without further imaging. Figure 5a–d, e–h and i–l present the vertical view, *x*-axis and *y*-axis cross-section views of the PSFs, respectively. As shown in Figure 5a, the target point is in the center of the imaging plane. Apparently, the grid cell containing scattering information shows distinct spike pulses, which is shown in Figure 5e,i. From Figure 5b to Figure 5d, the BP imaging results become more and more randomly with the increasing phase-modulation ranges. However, when the phase-modulation range is [−π, π], the PSF image becomes blurred and is difficult to detect the target point. As for Figure 5c, firstly, the BP projecting result shows the right position of the target, which is helpful for the EV extraction of BP-TCAI. Secondly, the random condition around the target point provides enough arbitrary measurement modalities for high-resolution imaging. Therefore, to compare the imaging performance of traditional TCAI, GM-TCAI and BP-TCAI, we set the phase-modulation range as [−0.5π, 0.5π], which strike a and high resolution.

### 3.2. Range Profile Analysis

**Figure 6:**
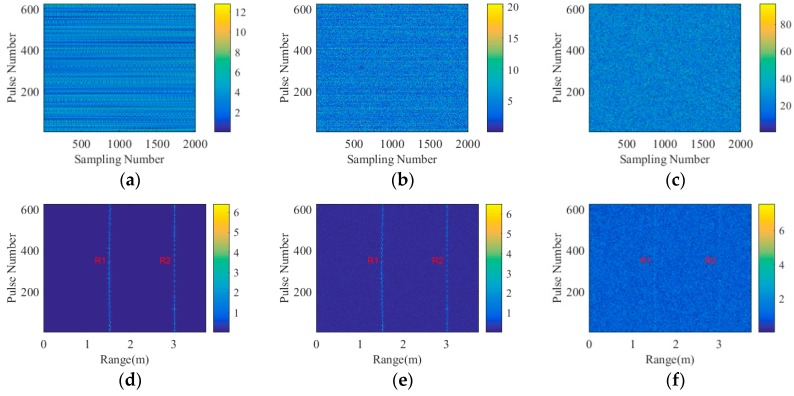
Original back signal under (**a**) 30 dB, (**b**) 0 dB and (**c**) −30 dB, respectively. Range profile under (**d**) 30 dB, (**e**) 0 dB and (**f**) −30 dB, separately. Herein, R1 and R2 describe the imaging planes 1 and 2 at 1.5m and 3m, respectively.

As shown in Table 1, the number of the coded-aperture array elements is 625 and the sampling times within a pulse is 2000. Therefore, the coded-aperture receives 625 pulses with the length of 2000. To obtain the range profiles of the echo signals, the 625 pulses are processed by IFT operation according to Equation (13). Figure 6a–c presents the original 625 pulses under 30 dB, 0 dB and −30 dB, respectively, while Figure 6d–f shows the corresponding range profiles. R1 and R2 are used to describe the imaging planes 1 and 2 at 1.5 m and 3 m, respectively. The echo signals become chaotic from Figure 6a–c with the decreasing SNRs. Under 30 dB and 0 dB, Figure 6d,e clearly presents the range positions of R1 and R2, separately. Although when the SNR declines to −30 dB, Figure 6f still shows two ambiguous lines at the positions of R1 and R2. Therefore, we can extract the related columns of R1 and R2 and then project them to the corresponding imaging planes by BP method, which is introduced in detail in Section 2.3.1.

### 3.3. Projection Results of BP

**Figure 7:**
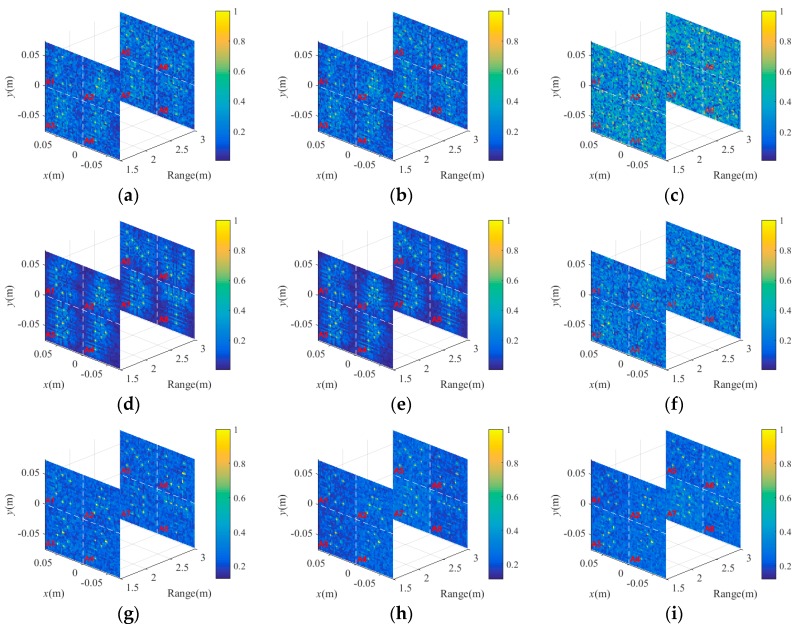
The BP projection results with [−0.5π, 0.5π] phase modulation under (**a**) 30 dB, (**b**) 0 dB and (**c**) −30 dB, respectively. The BP projection results without modulation under (**d**) 30 dB, (**e**) 0 dB and (**f**) −30 dB, respectively. The GM enhanced BP projection results with [−0.5π, 0.5π] phase modulation under (**g**) 30 dB, (**h**) 0 dB and (**i**) −30 dB, respectively. The “C,” “A,” “B” and “P” shape targets are distributed in R1, while “N,” “U,” “D” and “T” shape targets are located in R2. The “CABP” and “NUDT” denote coded aperture using BP and National University of Defense Technology, respectively. R1 contains four subareas named as A1–A4 and R2 has four subareas marked as A5–A8.

According to Equation (13), the range-profile signals in Figure 6d–f are projected into R1 and R2, which are shown in Figure 7a–c. Besides, Figure 7d–f illustrates the projection results without modulation. The “C,” “A,” “B” and “P” shape targets are distributed in R1, while “N,” “U,” “D” and “T” shape targets are located in R2. The “CABP” and “NUDT” denote coded aperture using BP and National University of Defense Technology, respectively. The eight subareas of R1 and R2 are named as A1–A8. When the SNRs are 30 dB and 0 dB, both the BP projection results with and without modulation can display the basic profile of the 3D target. Although the projection results with modulation are a little blurred, the seemed ambiguous images actually contain arbitrary coding information, which can be exploited by BP-TCAI. In contrast, BP-TCAI cannot achieve further imaging with the projection results without modulation in Figure 7d–f. Under −30 dB, the SNR is too low for both Figure 7c,f, to resolve the scattering distribution of the target. However, the GM method used in GM-TCAI^19^ can also be applied into BP-TCAI to detect the primary target positions. With the GM tool, regardless of the SNRs, Figure 7g–i show the scattering points of the target in the right positions, which can be used for EV extraction.

### 3.4. Imaging Results Analysis

**Figure 8:**
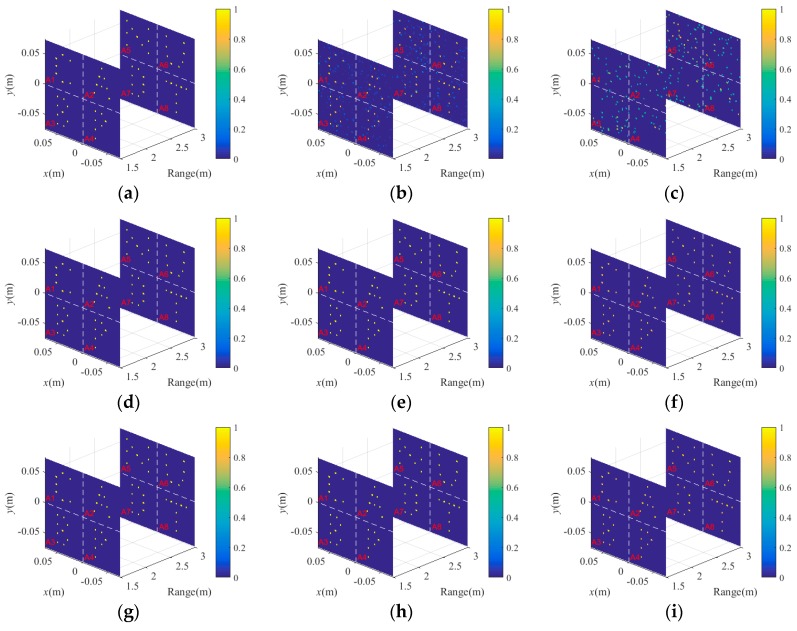
The imaging results of traditional TCAI under (**a**) 30 dB, (**b**) 0 dB and (**c**) −30 dB, respectively. The imaging results of GM-TCAI under (**d**) 30 dB, (**e**) 0 dB and (**f**) −30 dB, respectively. The imaging results of BP-TCAI under (**g**) 30 dB, (**h**) 0 dB and (**i**) −30 dB, respectively.

As shown in Figure 7, the projection results are divided to construct the EVs of different subareas via Equation (14). According to the extracted EVs and Equations (16)–(20), the corresponding RSMs are built for high-resolution imaging of BP-TCAI. To verify the superiority of BP-TCAI, Figure 8 compares the 3D imaging results of BP-TCAI and the previous algorithms, including traditional TCAI and GM-TCAI. The imaging models of traditional TCAI, GM-TCAI and BP-TCAI are based on Equations (5), (9) and (21), respectively. The imaging procedures of the three imaging methods are described in Table 1, Table 2 and Table 3, separately. Figure 8a–c presents the imaging results of tradition TCAI under 30 dB, 0 dB and −30 dB, respectively. Moreover, Figure 8d–f describes the imaging results of GM-TCAI, while Figure 8g–i show the imaging results of BP-TCAI under different SNRs. As shown in Figure 8a,d,g, when the SNR is 30 dB, all the three TCAI methods can reconstruct the 3D target. For 0 dB condition presented in Figure 8b,e,h, both BP-TCAI and GM-TCAI reconstruct the precise target while the traditional TCAI resolves the target with some background noise. When the SNR is greater than or equal to 0 dB, all the three imaging methods can reconstruct the 3D target regardless of the imaging performance. However, traditional TCAI resolves a cluster of disordered scattering points in the 3D imaging area under −30 dB, which is shown in Figure 8c. As for BP-TCAI and GM-TCAI, it is difficult to judge the image quality from Figure 8f,i, both of which resolve the clear 3D target. Therefore, we use RIE and PSI to compare the imaging performance of them.

**Figure 9:**
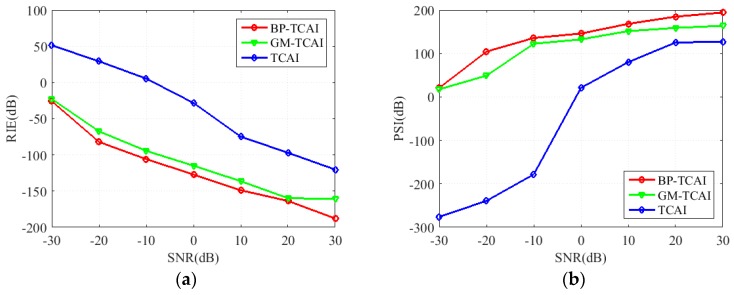
(**a**) RIE comparisons of TCAI, GM-TCAI and BP-TCAI, (**b**) PSI comparisons of TCAI, GM-TCAI and BP-TCAI.

Figure 9a,b presents the RIE and PSI comparisons of traditional TCAI, GM-TCAI and BP-TCAI. As described in the third paragraph of Section 3, the smaller RIE and the larger PSI denote better imaging performance. Illustrated by the blue lines in Figure 9, the largest RIE and the least PSI under different SNRs indicate that the imaging performance of traditional TCAI is the worst among the three imaging methods. The red and green lines in Figure 9 denote the imaging evaluations of BP-TCAI and GM-TCAI, respectively. Apparently, the imaging performance of BP-TCAI is slightly better than the GM-TCAI.

As shown in Table 4, the 3D imaging areas has two imaging planes, both of which contain 60 × 60 grid cells. Furthermore, each imaging plane has four subareas with 30 × 30 grid cells. When the number of sampling time and grid cells are the same, the RSM sizes of traditional TCAI, GM-TCAI and BP-TCAI are 7200 × 7200, 3600 × 3600 and 900 × 900, respectively. According to the theory analysis in Section 2.4, the computational complexity of TCAI relies mainly on the size of the RSM. Therefore, the computational burden of BP-TCAI is much less than traditional TCAI and GM-TCAI. Table 5 compares the runtime of the three imaging methods. Apparently, BP-TCAI achieves the fast imaging while traditional TCAI is the most time-consuming. Moreover, BP-TCAI can divide each imaging plane containing scattering information into more than four subareas. Therefore, the RSM of BP-TCAI can be further downsized to improve the computational efficiency. Actually, the shapes and sizes of the sub-areas can change according to the distribution of the target. Therefore, the computational time can be optimized with proper shapes and sizes. In conclusion, BP-TCAI is the most effective and efficient imaging method in contrast to traditional TCAI and GM-TCAI.

## 4. Conclusions

This paper proposes an imaging method of TCAI based on BP to reduce the computational burden and achieve high-resolution imaging for 3D imaging. Actually, BP helps to transforms the coding imaging from time domain into space domain, where different target parts can be reconstructed independently and simultaneously with higher SNR. Numerical experiments demonstrate that BP-TCAI is an effective and efficient imaging method. Furthermore, the BP-TCAI can also be seen as a combination of the traditional SAR imaging and the coding strategy. Therefore, BP-TCAI not only can be used to improve the imaging performance of TCAI but also provide a prospective approach for traditional SAR. Though this proposed imaging method is verified to be more effective and efficient than the previous works, there are still some limitations that can be studied by other researchers. Firstly, the imaging performance of BP-TCAI is only slightly better GM-TCAI, which is the best improved TCAI proposed in our previous paper. With the same reconstruction algorithm, the imaging performance is mainly decided by the SNR. The SNR of BP-TCAI can be improved during the pulse compression and projection processes while the SNR of GM-TCAI can also be improved by pulse compression. Therefore, the SNR or the imaging performance is only improved in a certain degree by the projection operation of BP. Secondly, the imaging efficiency can still be improved even BP-TCAI is more efficient than before. According to the basic principle of SAR imaging, BP algorithm is an effective imaging method but consumes much time in the projection procedure. In the future work, some other useful imaging theories rather than BP can be used to project the signals into space areas with more effective and efficient approach.

## Figures and Tables

**Table 1:** Imaging procedure of traditional terahertz coded-aperture imaging (TCAI).

Requirement	A Computer, A Transmitter and A Coded Aperture.
Imaging process	Step 1: Obtain the echo vector (EV) Sr by the following procedures. (1) The computer controls the transmitter to send signal. (2) Controlled by the computer, the coded aperture randomly modulates the transmitting signal. (3) The single detector receives the echo signal, which carries the 3D target information.
	Step 2: Construct the reference-signal matrix (RSM) according to Equation (6).
	Step 3: Reconstruct the estimated scattering-coefficient vector (SCV) $\hat{\beta }$ via Equation (5)
Output	Return the TCAI imaging result $\hat{\beta }$.

**Table 2:** Imaging procedure of geometric measures (GM)-TCAI.

Input	The Time-Domain EV Sr.
Imaging process	Step 1: *parfor x* = 1:X (parfor denotes the for loop in parallel, X means the total imaging-plane numbers) (1) Extract EV $FSr_{x}$ from FSr, which is transformed from Sr via Equation (7). Besides, the row positions of $FSr_{x}$ in FSr is indexed as $r_{x}$. (2) According to the detailed processes and Equation (8) in Section 2.2.2, construct the RVM $FS_{x}$ corresponding to $FSr_{x}$. (3) Reconstruct ${\overset{⌢}{\beta }}_{x}$ via Equation (9) *end*
	Step 2: Obtain the 3D imaging result ${\hat{\beta }}_{GM}$ in combination of ${\overset{⌢}{\beta }}_{x},\;x=1:X$.
Output	Return the GM-TCAI imaging result ${\hat{\beta }}_{GM}$.

**Table 3:** Imaging procedure of back projection (BP)-TCAI.

Requirement	A Computer and a Coded-Aperture Array Transceiver.
Imaging process	Step 1: Obtain the time domain echo signal by the following procedures. (1) The computer controls the single transmitter to send signals. (2) Multiple coded-aperture detectors randomly modulate and receive the echo signals. (3) The modulated echo signals are transported into the computer for imaging.
	Step 2: *parfor x* = 1:X *parfor* a = 1:A (A describes the imaging-area numbers in imaging plane *x*) (1) Extract SD-EV $BSr_{x}$ from $BSr_{x}$, which is deduced from Equation (15). Besides, the row positions of $BSr_{x_{a}}$ is indexed as $r_{x_{a}}$. (2) Construct the RVM of BP-TCAI $BS_{x_{a}}$ corresponding to $BSr_{x_{a}}$ according to the detailed processed in Section 2.3.2 and Equations (16)–(20). (3) Reconstruct ${\overset{⌢}{\beta }}_{x_{a}}$ via Equation (21). *end* *end*
	Step 3: Obtain the 3D imaging result ${\hat{\beta }}_{BP}$ in combination of ${\overset{⌢}{\beta }}_{x_{a}},\;x=1:X,a=1:A$.
Output	Return the BP-TCAI imaging result ${\hat{\beta }}_{BP}$.

**Table 4:** Primary parameters used in the experiments.

Parameter	Value
Center frequency (*f_c_*)	340 GHz
Bandwidth (B)	20 GHz
Pulse Width (*T_p_*)	100 ns
Size of the coded aperture	0.5 m × 0.5 m
Number of coded-aperture array elements	25 × 25
Range of Scene 1	1.5 m
Range of Scene 2	2 m
Range of Scene 3	2.5 m
Range of Scene 4	3 m
Size of the grid cell	0.0025 m × 0.0025 m

**Table 5:** Runtime comparisons of BP-TCAI, GM-TCAI and traditional TCAI.

BP-TCAI	GM-TCAI	TCAI
2.4258	8.7253	16.8327
